# Diagnostic Accuracy of the Primary Care PTSD for DSM-5 Screen (PC-PTSD-5) in Demographic and Diagnostic Subgroups of Veterans

**DOI:** 10.1007/s11606-024-08719-5

**Published:** 2024-03-20

**Authors:** Quyen Q. Tiet, Thien-Nha Tiet

**Affiliations:** 1 California School of Professional Psychology at Alliant International University, PhD Clinical Psychology Program, San Francisco Bay Area Campus, Emeryville, CA USA; 2 National Center for PTSD, Dissemination and Training Division, VA Palo Alto Health Care System, Menlo Park, CA USA; 3 Department of Psychology, University of California, San Diego, CA USA

## Abstract

**Background:**

The Primary Care PTSD Screen for DSM-5 (PC-PTSD-5) is a screening instrument designed to identify patients with probable PTSD and is mandated to be used in VA primary care settings. However, validation of the diagnostic accuracy of the instrument is lacking in various demographic and diagnostic groups.

**Objective:**

To evaluate the diagnostic accuracy of the PC-PTSD-5 in demographically and diagnostically stratified groups of VA primary care patients.

**Design, Setting, and Participants:**

Based on a sample of 519 VA primary care patients (40.61% response rate), the PC-PTSD-5 was evaluated against a criterion measure, examining its sensitivity, specificity, and other diagnostic properties. Evaluations were conducted in demographically stratified patient groups, including gender, age, race/ethnicity, marital status, and education, as well as diagnostically stratified groups, in patients with and without a depression, alcohol use, and drug use disorder.

**Main Measures:**

The MINI-International Neuropsychiatric Interview was the criterion measure against which the PC-PTSD-5 was evaluated.

**Key Results:**

Among the 10 demographically stratified groups of patients, the PC-PTSD-5 demonstrated a sensitivity between 81.25% (95% *C.I.*: 54.35 – 05.95) and 100% (95% *C.I.*: 63.06 – 1000) and its specificity ranged from 80.54% (95% *C.I.*: 74.70 – 85.55) to 88.31% (95% *C.I.*: 83.45 – 92.15). Among the 6 diagnostically stratified groups of patients (with and without depression, alcohol use, and drug use disorders), the PC-PTSD-5 exhibited a sensitivity between 88.89% (95% *C.I.*: 65.29 – 98.62) and 95.92% (95% *C.I.*: 86.02 – 99.50), and its specificity varied from 60.00% (95% *C.I.*: 40.60 – 77.34) to 88.14% (95% *C.I.*: 84.50 – 91.19).

**Conclusions:**

The PC-PTSD-5, suitable for a diverse range of VA primary care patients, shows satisfactory sensitivity and specificity across various demographic and diagnostic groups. Healthcare providers should be cautious of false positives in patients with depression or substance use disorders, given the potential symptom overlap with PTSD.

Posttraumatic stress disorder (PTSD) is a debilitating psychiatric condition that can significantly impair an individual's functioning and overall well-being.^1^ PTSD is one of the most prevalent (10-20%) mental disorders observed among veterans following their return from war.^2,3^ Veterans with PTSD are more likely to have various medical co-morbidities^4^ and develop depression, anxiety, and other psychiatric disorders longitudinally.^5^ Additionally, PTSD is linked to heightened medical disease burden and health care utilization and cost in veterans.^6^ However, PTSD often goes undetected, undiagnosed and untreated in primary care.^7^ In both US general populations and military veterans, studies indicate lower treatment utilization among certain racial and ethnic minority groups compared to non-Hispanic White individuals.^8^ Timely identification and appropriate intervention for PTSD are critical in primary care settings,^9,10^ because patients with PTSD often present in primary care clinics for physical health problems.^11^

The Primary Care PTSD Screen for DSM-5^12^ is an updated screening instrument from the PC-PTSD^13^ for DSM-IV, and it has been mandated to be used routinely in the VA primary care setting.^14^ The updated PC-PTSD-5 is a 5-item screening instrument using the DSM-5 diagnostic criteria. The screen was designed to identify individuals likely to have PTSD and to be easily administered and interpreted by primary care providers, enabling prompt recognition and referral for specialized mental health services.^12^ Patients who screen positive should be addressed and considered for referral to VA PTSD specialty settings for further evaluation and treatment.^15^ Diagnostic accuracy properties, including sensitivity and specificity, of the PC-PTSD-5 were examined against a modified version of the PTSD module of the MINI-International Neuropsychiatric Interview on a sample of Veterans recruited from VA primary care clinics. The PC-PTSD-5 screen demonstrated satisfactory psychometric properties in the general primary care veteran population to identify individuals with probable PTSD, with 95% sensitive and 85% specific.^12^ It has been widely used and has been validated again in the veteran,^16^ and civilian populations.^17,18^ In addition, it has been translated and validated in other languages internationally.^19,20^ However, its validation across various racial and ethnic groups, other demographic groups, as well as various diagnostic groups is still unexplored.

An essential reason for validating the PC-PTSD-5 screen across diverse racial and ethnic populations is to ensure its reliability and validity across a wide spectrum of patients. If the instrument proves less sensitive in a particular subgroup of patients, individuals within that subgroup with PTSD may be less likely to have their condition detected, assessed, and followed up with necessary intervention. Racial and ethnic minority military veterans were found to experience greater exposure to traumatic events and have higher rate of PTSD.^21^ Hispanic/Latino and Black military members with PTSD reported more PTSD symptoms than their non-Hispanic White counterparts who also had PTSD.^22^ Historically, racial and ethnic minorities have faced disparities in mental healthcare access and quality, and they may experience unique barriers to seeking help for mental health concerns.^23^ By validating the PC-PTSD-5 screen in diverse racial and ethnic groups, we can determine whether the screen effectively captures PTSD symptoms of patients across different cultural backgrounds, thus enhancing our knowledge of its applicability and accuracy in various patient groups.

Moreover, it is not uncommon for individuals with PTSD to experience comorbid conditions, especially depression, and substance use disorders (SUDs).^24,25^ Simpson et al.,^26^ based on the NESARC-III dataset, showed that among individuals with co-occurring PTSD and SUD, only 25% received any PTSD treatment in the past year. These comorbidities influence symptom presentation and may influence the accuracy of the PC-PTSD-5 screening instrument.^27^ Therefore, the validation of the PC-PTSD-5 screen in various diagnostic groups is essential. Through an evaluation of the PC-PTSD-5 screen in diagnostic groups that encompass individuals both with and without depression and SUD, providers can gauge its sensitivity, specificity, and other psychometric properties when employed as a tool for PTSD screening in the primary care settings.

In summary, there is a considerable lack of validation of the PC-PTSD-5 screening instrument across diverse demographic and diagnostic groups. This study sought to evaluate the sensitivity, specificity, and other psychometric characteristics of the PC-PTSD-5 within various demographic categories, encompassing race/ethnicity, gender, age, marital status, and education. Additionally, it aimed to assess the PC-PTSD-5 in distinct diagnostic groups, including patients with and without depression, alcohol, and drug use disorders. The findings from this research are expected to provide crucial guidance on the application of this instrument within each subgroup of VA primary care patients.

## Methods

### Procedure

This study used the same dataset that Prins et al. (2016) used to develop the PC-PTSD-5. The data were collected as part of the Drug Screen for Primary Care Patients Study,^28^ which constituted a broader project with the primary objective of developing and validating a drug screening tool suitable for VA primary care settings. The dataset has been used to develop and validate a number of drug screening instruments, including the Screen of Drug Use (SoDU),^28^ and its variants to target specific drugs, including opioid,^29^ cannabis,^30^ and stimulants^31^ as well as the two-item Drug Abuse Screening Test (DAST-2),^32^ and the two-item Alcohol, Smoking, and Substance Involvement Screening Test (ASSIST-Drug).^33^ The Drug Screen Study, encompassing all aspects of this study, received approval from the local Institutional Review Board.

Data collection for this study took place from September 2012 through December 2013.^28^ The research team recruited patients from the waiting areas of one of two VA primary care clinics. All current primary care patients at either clinic were eligible for participation, with exclusion criteria related to the ability to provide informed consent. Trained research staff conducted interviews with participants.

### Participants

In this study, out of the 1,278 participants approached, 519 (40.61%) participated, comprising 498 (95.95%) men. The participants’ ages ranged from 23.8 to 89.0, with a mean age of 63.2 (SD = 12.59). The majority were non-Hispanic White individuals (54.91%), with 77 (14.84%) Black, 46 (8.86%) Native American or Alaska Native, 35 (6.74%) Asian, and with 99 (19.08%) identified as Hispanic of any race.

### Measures

#### PTSD Screen

The Primary Care PTSD Screen for DSM-5 (PC-PTSD-5)^12^ is a 5-item screening instrument designed for the identification of PTSD in the VA primary care settings. The five items assess the presence of nightmares, avoidance symptoms, arousal and reactivity symptoms, numbing, and negative alterations in mood and cognitions. Scoring involves summing the yes/no responses. To maximize sensitivity, a cut-point of 3 was found to be 95% sensitive when examined against the MINI-International Neuropsychiatric Interview (MINI)^34^ in a sample of VA primary care veterans.^12^

#### Criterion Measure

The MINI-International Neuropsychiatric Interview (MINI)^34^ is a brief, structured, diagnostic interview designed to be administered by trained interviewers. It has good concordance with both the Composite International Diagnostic Interview^35^ and the Structured Clinical Interview for DSM.^36^

During the study period, validation studies for clinical interviews on DSM-5 PTSD criteria had not been published. Consequently, a modified version of the DSM-IV PTSD module from the MINI was employed to evaluate DSM-5 criteria. Consistency is maintained because this modified version of the PTSD module is identical to the one used by Prins et al. (2016)^12^ in developing and validating the PC-PTSD-5, with both studies utilizing the same dataset. In summary, the probe was adjusted for the DSM-5, and questions were added and modified to be consistent with the DSM-5.^12^

The modified MINI was administered by trained researchers. The interviews were audio recorded and randomly selected (11.1%) to check interrater reliability. Reliability at the item level was maintained at κ > .95, and perfect interrater reliability was maintained for PTSD diagnosis.^28^

#### Alcohol and Drug Use Disorders

The MINI^34^ was employed to evaluate both alcohol use and drug use disorders. DSM-IV assessment provided a diagnosis of substance abuse or dependence. Substance use disorder in DSM-5 combines the DSM-IV categories of substance abuse and substance dependence into a single category of substance use disorder. Assessment of alcohol abuse and alcohol dependence disorders were combined into a single category of alcohol use disorder for this study. Regarding drug use disorders, the MINI encompassed both illicit and prescribed medications across eight major categories of substances: stimulants, cocaine, narcotics, hallucinogens, inhalants, marijuana, tranquilizers, and miscellaneous. Symptoms of drug use were assessed, and a diagnosis of drug abuse or dependence disorder was combined into a single category of a drug use disorder for DSM-5 in this study.

#### Depression

The Patient Health Questionnaire (PHQ-9––)^37^ was used to assess depression. The cut-off score of 10 or above has been validated and recommended to maximize sensitivity and specificity.^38^ Using this threshold, the sample was stratified into subgroups of patients with and without depressive disorder.

### Data Analysis

The diagnostic accuracy of the PC-PTSD-5 was evaluated against the MINI within various stratified patient groups, including gender, age, race/ethnicity, marital status, education, and presence or absence of alcohol use disorder, drug use disorder, and depression disorder (see Table 1). The study computed five diagnostic metrics for the PC-PTSD-5, including sensitivity, specificity, accuracy, positive likelihood ratio, and negative likelihood ratio. Sensitivity (true positive / true positive + false negative) is a measure of a diagnostic test's ability in identifying individuals with the condition or disease of interest, while specificity (true negative / false positive + true negative) gauges its ability to correctly identify those without the condition. Accuracy assesses the overall performance of a test in classifying both positive and negative cases. The positive likelihood ratio (LR+) indicates the increased likelihood of a positive test result in individuals with the condition compared to those without it, with a higher score indicating a better test. Conversely, the negative likelihood ratio (LR-) signifies the heightened likelihood of a negative test result in individuals with the condition versus those without it, with a lower score signifying a more favorable test.

**Table 1:** Diagnostic properties of the Primary Care PTSD for DSM-5 Screen within Demographic and Diagnostic Subgroups of VA Patients.

Group	N	SENS [95% CI]	SPEC [95% CI]	Accuracy	LR+ [95% CI]	LR- [95% CI]
Total Sample	519	94.03 [85.41 – 98.35]	84.51 [80.84 – 87.72]	85.74 [82.30 – 88.64]	6.07 [4.85 – 7.59]	0.07 [0.03 - 0.18]
Male	498	93.22 [83.54 - 98.12]	84.51 [80.78 – 87.77]	85.54 [82.14 – 88.51]	6.02 [4.79 – 7.57]	0.08 [0.03 - 0.21]
Female	21	100 [63.06 – 100.00]	84.62 [54.55 – 98.08]	90.48 [69.62 - 98.83]	6.50 [1.82 – 23.26]	0.00
Age, Younger (<65)	266	95.56 [84.85 - 99.46]	80.54 [74.70 – 85.55]	83.08 [78.03 – 87.38]	4.91 [3.73 – 6.47]	0.06 [0.01 - 0.21]
Age, Older (65+)	253	90.91 [70.84 - 98.88]	88.31 [83.45 – 92.15]	88.54 [83.95 – 92.19]	7.78 [5.33 – 11.35]	0.10 [0.03 - 0.39]
Non-Hispanic White	285	96.00 [79.65 – 99.90]	84.62 [79.65 – 88.78]	85.61 [80.99 – 89.47]	6.24 [4.64 – 8.39]	0.05 [0.01 - .032]
Ethnic Minority	233	92.86 [80.52 - 98.50]	84.29 [78.34 – 89.14]	85.84 [80.69 – 90.05]	5.91 [4.21 – 8.30]	0.08 [0.03 - 0.25]
Married/Partnered	206	92.31 [74.87 - 99.05]	86.11 [80.18 – 90.81]	86.89 [81.51 – 91.18]	6.65 [4.54 – 9.72]	0.09 [0.02 - 0.34]
Single/Sep/Div/Wid	313	95.12 [83.47 - 99.40]	83.46 [78.50 – 87.67]	84.98 [80.54 – 88.75]	5.75 [4.36 – 7.58]	0.06 [0.02 - 0.23]
≤ HS	114	81.25 [54.35 – 95.95]	85.71 [77.19 – 91.96]	85.09 [77.20 – 91.07]	5.69 [3.32 – 9.75]	0.22 [0.08 - 0.61]
Some College +	277	98.04 [89.55 - 99.95]	84.18 [79.95 – 87.82]	85.93 [82.15 – 89.16]	6.20 [4.86 – 7.91]	0.02 [0.00 - 0.16]
No Alcohol Use Disorder	444	93.75 [82.80 – 98.69]	86.11 [82.31 – 89.36]	86.94 [83.44 – 89.93]	6.75 [5.23 – 8.72]	0.07 [0.02 - 0.22]
Alcohol Use Disorder	75	94.74 [73.97 - 99.87]	73.21 [59.70 – 84.17]	78.67 [67.68 – 87.29]	3.54 [2.26 – 5.52]	0.07 [0.01 - 0.49]
No Drug Use Disorder	472	94.00 [83.45 – 98.75]	86.26 [82.60 – 89.40]	87.08 [83.71 – 89.97]	6.84 [5.33 – 8.77]	0.07 [0.02 - 0.21]
Drug Use Disorder	47	94.12 [71.31 – 99.85]	60.00 [40.60 – 77.34]	72.34 [57.36 – 84.38]	2.35 [1.49 – 3.71]	0.10 [0.01 – 0.67]
No Depression	406	88.89 [65.29 – 98.62]	88.14 [84.50 – 91.19]	88.18 [84.63 – 91.15]	7.50 [5.46 – 10.29]	0.13 [0.03 – 0.47]
Depression	110	95.92 [86.02 – 99.50]	60.66 [47.31 – 72.93]	76.36 [67.32 – 83.94]	2.44 [1.78 – 3.35]	0.07 [0.02 – 0.27]

SENS = sensitivity; SPEC = specificity; LR+ = positive likelihood ratio; LR- = negative likelihood ratio; Single/Sep/Div/Wid = single, separated, divorced, or widowed; ≤ HS = high school or lower education level; Some College + = some college or higher education level

## Results

In the overall sample of 519 participants, the PC-PTSD-5 showed a sensitivity of 94.03% and specificity of 84.51% (Table 1). It had an accuracy of 85.74%. Additionally, the positive likelihood ratio was 6.07, and a negative likelihood ratio was 0.07.

Among the 10 demographically stratified groups of patients, the PC-PTSD-5 demonstrated a sensitivity between 90.91% and 100% in 9 groups of patients, except for a sensitivity of 81.25% in patients who completed high school or had a lower education level. Among these 10 stratified groups of patients, its specificity ranged from 80.54% to 88.31%. Its accuracy spanned from 83.08% to 90.48%.

Among the 6 diagnostically stratified groups of patients, the PC-PTSD-5 exhibited a sensitivity of 93.75% or higher in 5 groups of patients, except for a sensitivity of 88.89% among the stratified group of patients who did not have a depressive disorder. The PC-PTSD-5 had higher specificity rates among stratified groups of patients without a depression or substance use disorder than its rates among stratified groups of patients with the disorders examined. Among stratified groups of patients without a depression or substance use disorder, the PC-PTSD-5 exhibited a specificity ranging from 86.11% to 88.16%. However, among those stratified groups of patients with a depression or substance use disorder, the specificity of the PC-PTSD-5 varied between 60.00% among patients with a drug use disorder and 73.21% in patients with an alcohol use disorder.

## Discussion

This study presents an essential investigation into the diagnostic properties of the PC-PTSD-5 within demographically and diagnostically stratified groups of VA primary care patients. Specifically, it delves into the PC-PTSD-5's diagnostic properties across a wide spectrum of patient cohorts, encompassing factors including age, gender, race and ethnicity, educational background, and marital status. Moreover, this study also evaluates the PC-PTSD-5's performance in stratified groups of patients both with and without concurrent conditions, including depression disorder, alcohol use disorder, and drug use disorders. Among the 10 demographically stratified groups of patients (gender, age, race/ethnicity, relationship status, and education), the PC-PTSD-5 demonstrated a sensitivity between 81.25% and 100% and its specificity ranged from 80.54% to 88.31%. Among the 6 diagnostically stratified groups of patients (with and without depression, alcohol use, and drug use disorders), the PC-PTSD-5 exhibited a sensitivity between 88.89% and 95.92%, and its specificity varied from 60.00% to 88.14%.

The primary finding of this study emphasizes the PC-PTSD-5's consistent high sensitivity across diverse patient groups, showcasing its robust capability as a versatile diagnostic screen to effectively identify potential cases of PTSD. This holds true regardless of variations in patient diagnostic or demographic profiles, encompassing individuals from racial and ethnic minority backgrounds. This reassurance is crucial for decision-makers considering the increased use of this screener within this population. Increased utilization of the PC-PTSD-5 among racial and ethnic minority individuals holds the potential to enhance the detection, assessment, and treatment of PTSD, ultimately contributing to the reduction of service disparities for this population.

The PC-PTSD-5, mandated for routine use in primary care by the VA, is pivotal in advancing healthcare delivery. It plays a crucial role in identifying individuals with undiagnosed and untreated PTSD, offering a streamlined and standardized approach to screening. This proactive use of the PC-PTSD-5 enhances the likelihood of early detection and referral, contributing to improved outcomes for diverse patient populations. Through systematic screening and timely interventions, the PC-PTSD-5 has the potential to help alleviate the underdiagnosis and undertreatment of PTSD, thereby enhancing the overall quality of life for diverse affected individuals. In essence, this instrument could act as a catalyst for positive changes, promoting more efficient and equitable healthcare delivery for those grappling with post-traumatic stress disorder in primary care settings, including racial and ethnic minority populations.

Notably, the sensitivity of the test is slightly reduced in patients with a lower education level compared to those with a higher education level. Among the 114 individuals with lower education in this sample, 104 completed high school. Prins et al.^12^ reported that the measure's reading level was at a Flesch-Kincaid grade level of 6.5, suggesting reading level may not solely contribute to the difference. The challenges of understanding and accurately responding to the screen at the conceptual level might hinder individuals with lower educational backgrounds, contributing to the compromised sensitivity of the measure. Future research should prioritize enhancing the sensitivity of the PC-PTSD-5 for individuals with lower educational levels, potentially through qualitative studies or conducting focus groups with individuals with lower educational levels to revise the instrument’s items to elicit accurate responses as intended.

Additionally, it's worth noting that the specificity of the PC-PTSD-5 tends to be lower in patients with comorbid depression or substance use disorders compared to those without these conditions. In simpler terms, the screening tool may incorrectly categorize individuals without PTSD as positive, incorrectly suggesting they have the condition when they, in fact, do not, especially among patients with depression and substance use disorders. Consequently, healthcare providers should exercise caution when evaluating patients with depression and substance use disorders, as they may exhibit symptoms that mimic PTSD,^27,39^ potentially leading to a false-positive result on the PC-PTSD-5 screening. Therefore, the PC-PTSD-5 screener should not be used to diagnose PTSD, especially among patients with co-occurring depression or SUD disorders. Follow-up clinical assessment should always be used for a positive screen. Healthcare systems should also consider this factor when implementing universal screening in their healthcare settings, as such false-positive results may necessitate unnecessary further assessments and incur additional healthcare costs.

The current study relied on a convenient sample primarily composed of older, White, primary care VA patients, which may limit the generalizability of the findings to all VA patient groups. While it is noteworthy that this sample's demographics align with the broader VA patient population,^40^ further research is essential to validate the present results, especially among women, and individuals with alcohol and drug use disorders. Additionally, there remains uncertainty regarding the PC-PTSD-5's performance in demographic and diagnostic subgroups of non-VA primary care patient populations. It is also important to note that the study's criteria for diagnosis did not rely on assessments by licensed clinicians. Lastly, due to constraints posed by sample limitations, the study was unable to explore the diagnostic properties of the PC-PTSD-5 among subgroups within broader categories, including various age subgroups and diverse racial and ethnic groups. In addition, subgroups of sexual minorities were not examined in the study.

Despite these limitations, results from the present study strongly support the PC-PTSD-5 for routine use within the VA primary care settings, as it is currently mandated.^14^ The routine use of the PC-PTSD-5 as a universal screening tool holds benefits for patients. It enables the early detection of PTSD to facilitate timely intervention and treatment. By normalizing mental health discussions in primary care settings, it helps reduce stigma, encouraging patients to seek help without fear of judgment.^41^ Early intervention not only enhances patients' quality of life but also prevents the progression of the disorder and development of comorbid conditions, hence reducing the complexity and cost of treatment.

In summary, the PC-PTSD-5 emerges as a valuable screening instrument suitable for a wide range of VA primary care patients. The PC-PTSD-5 maintains satisfactory sensitivity as well as specificity levels across all demographic categories, making it a valuable tool for the primary care system. Additionally, it has good to excellent sensitivity across various demographic and diagnostic groups, ensuring its utility in identifying potential cases of PTSD in all patients.
